# Tendência temporal e perfil clínico-epidemiológico da coinfecção por leishmaniose visceral e HIV em estados brasileiros entre 2009 e 2024

**DOI:** 10.1590/0102-311XPT163425

**Published:** 2026-07-06

**Authors:** Marina França, Mirella Ferreira da Cunha Santos, Juceli Gonzalez Gouveia, Leandro Antero

**Affiliations:** 1 Universidade Estadual do Mato Grosso do Sul, Dourados, Brasil.

**Keywords:** Leishmaniose Visceral, HIV, Coinfecção, Políticas de Saúde, Visceral Leishmaniasis, HIV, Coinfection, Health Policies, Leishmaniasis Visceral, VIH, Coinfección, Políticas de Salud

## Abstract

O objetivo foi analisar a tendência temporal e o perfil clínico-epidemiológico da coinfecção por leishmaniose visceral (LV) e HIV no Brasil entre 2009 e 2024. Estudo ecológico de série temporal e transversal descritivo, baseado em dados do Sistema de Informação de Agravos de Notificação (SINAN) de oito estados com maior incidência de LV: Bahia, Ceará, Maranhão, Minas Gerais, Mato Grosso do Sul, Pará, São Paulo e Tocantins. As taxas anuais de coinfecção foram calculadas pela razão entre o número de casos notificados de coinfecção LV/HIV e a população residente no respectivo estado no mesmo ano, multiplicada por 100 mil. A tendência temporal foi avaliada por regressão segmentada, estimando a variação percentual anual (APC, acrônimo em inglês). Realizaram-se análises de correlação (Spearman), associações categóricas (qui-quadrado de Pearson), diferenças de médias (t de Student) e regressão logística binária (*odds ratio* - OR) para sintomas. Verificou-se tendência crescente da taxa de coinfecção na Bahia (APC = 4,2), Mato Grosso do Sul (APC = 19,7), Pará (APC = 10,4) e Tocantins (APC = 8,4). Ceará e Minas Gerais apresentaram crescimento seguido de queda. Observou-se correlação positiva moderada entre casos de LV e coinfecção (ρ = 0,313; p < 0,001). Coinfectados tinham maior média de idade (37,9 *vs*. 27,3 anos; p < 0,001) e predomínio masculino (72,4% *vs*. 59,8%). A raça parda foi prevalente, com maior proporção de pretos entre os coinfectados. Sintomas associados à coinfecção incluíram febre (OR = 2,44), icterícia (OR = 1,69) e edema (OR = 1,56); emagrecimento, hepatomegalia e infecção associada reduziram a chance. A coinfecção LV/HIV apresenta padrões heterogêneos, com tendências crescentes nos estados endêmicos e segmentos temporais distintos entre Unidades Federativas. Os achados reforçam a necessidade de estratégias integradas de vigilância, diagnóstico e tratamento.

## Abstract (en)

This study aimed to analyze temporal trends and the clinical-epidemiological profile of visceral leishmaniasis (VL)-HIV coinfection in Brazil between 2009 and 2024. This ecological time-series and descriptive cross-sectional study used data from the Brazilian Information System for Notifiable Diseases (SINAN, acronym in Portuguese) covering eight states with the highest VL incidence: Bahia, Ceará, Maranhão, Minas Gerais, Mato Grosso do Sul, Pará, São Paulo, and Tocantins. Annual coinfection rates were calculated as the ratio between the number of reported VL-HIV coinfection cases and the resident population of the respective state in the same year, multiplied by 100,000. Temporal trends were evaluated using segmented regression to estimate the annual percentage change (APC). Correlation analyses (Spearman), categorical associations (Pearson’s chi-squared test), differences in means (Student’s t-test), and binary logistic regression (odds ratio - OR) for symptoms were also performed. An increasing trend in coinfection rates was observed in Bahia (APC = 4.2), Mato Grosso do Sul (APC = 19.7), Pará (APC = 10.4), and Tocantins (APC = 8.4). Ceará and Minas Gerais showed an initial increase followed by a decline. A moderate positive correlation was observed between VL cases and coinfection (ρ = 0.313; p < 0.001). Coinfected individuals had a higher mean age (37.9 versus 27.3 years; p < 0.001) and were predominantly male (72.4% versus 59.8%). Mixed-race individuals were the most prevalent, with a higher proportion of Black individuals among coinfected patients. Symptoms associated with coinfection included fever (OR = 2.44), jaundice (OR = 1.69), and edema (OR = 1.56), whereas weight loss, hepatomegaly, and associated infection were associated with lower odds. VL-HIV coinfection shows heterogeneous patterns, with increasing trends in endemic states and distinct temporal segments across Federative Units. These findings reinforce the need for integrated strategies for surveillance, diagnosis, and treatment.

## Resumen

El objetivo del estudio fue analizar la tendencia temporal y el perfil clínico-epidemiológico de la coinfección por leishmaniasis visceral (LV) y VIH en Brasil entre 2009 y 2024.Tratase de un estudio ecológico de series temporales y transversal descriptivo basado en datos del Sistema de Información de Agravios de Notificación (SINAN) de ocho estados con mayor incidencia de LV: Bahía, Ceará, Maranhão, Minas Gerais, Mato Grosso do Sul, Pará, São Paulo y Tocantins. Las tasas anuales de coinfección se calcularon mediante la relación entre el número de casos notificados de coinfección LV/VIH y la población residente del estado correspondiente en el mismo año, multiplicada por 100.000. La tendencia temporal se evaluó mediante regresión segmentada, estimando la variación porcentual anual (APC, sigla en inglés). Se realizaron análisis de correlación (Spearman), asociaciones categóricas (chi cuadrado de Pearson), diferencias de medias (t de Student) y regresión logística binaria (*odds ratio* - OR) para los síntomas. Se observó una tendencia al alza en la tasa de coinfección en Bahía (APC = 4,2), Mato Grosso do Sul (APC = 19,7), Pará (APC = 10,4) y Tocantins (APC = 8,4). Ceará y Minas Gerais presentaron un crecimiento seguido de una caída. Se observó una correlación positiva moderada entre los casos de LV y la coinfección (ρ = 0,313; p < 0,001). Los coinfectados tenían una edad media más alta (37,9 frente a 27,3 años; p < 0,001) y predominaban los hombres (72,4% frente a 59,8%). La raza mestiza fue la más prevalente, con una mayor proporción de negros entre los coinfectados. Los síntomas asociados a la coinfección incluyeron fiebre (OR = 2,44), ictericia (OR = 1,69) y edema (OR = 1,56); la pérdida de peso, la hepatomegalia y la infección asociada redujeron la probabilidad. La coinfección LV/VIH presenta patrones heterogéneos, con tendencias crecientes en los estados endémicos y segmentos temporales distintos entre las Unidades Federativas. Los hallazgos refuerzan la necesidad de estrategias integradas de vigilancia, diagnóstico y tratamiento.

## Introdução

A leishmaniose visceral (LV) é uma zoonose de alta letalidade que persiste como importante problema de saúde pública no Brasil, especialmente em áreas de maior vulnerabilidade social ^1^. Classificada entre as doenças tropicais negligenciadas prioritárias pelo Ministério da Saúde e pela Organização Mundial da Saúde (OMS), a LV apresenta incidência concentrada nas regiões Nordeste e Centro-oeste, com maior número de casos em estados como Maranhão, Ceará, Bahia, Piauí e Mato Grosso do Sul ^2^ ^,^ ^3^.

Sua expansão para áreas urbanas reflete transformações ambientais, pobreza estrutural e fragilidades nos sistemas locais de vigilância e controle ^4^ ^,^ ^5^. Esse contexto de vulnerabilidade se sobrepõe à persistência da infecção pelo HIV, que mantém relevância epidemiológica no país e amplia o risco de sobreposição entre ambos os agravos. A coinfecção LV/HIV tende a ocorrer em contextos de pobreza estrutural e acesso limitado ao diagnóstico e ao tratamento, configurando um desafio adicional para os serviços de saúde ^6^ ^,^ ^7^. Além disso, essa coinfecção está associada a um grau aumentado de ativação imunológica, evidenciado por níveis elevados de citocinas inflamatórias, como IL-6 e outros marcadores de inflamação sistêmica ^8^, o que pode contribuir para uma maior morbidade, mortalidade e dificuldades no manejo clínico desses pacientes.

A coinfecção LV/HIV tem relevância clínica e epidemiológica reconhecida, sendo considerada uma condição definidora da aids segundo a OMS ^9^. Essa sobreposição agrava o curso clínico da doença, eleva a letalidade e impõe desafios terapêuticos adicionais, sobretudo em contextos de vulnerabilidade social e baixa cobertura assistencial. No Brasil, a distribuição dos casos de coinfecção é desigual entre os estados, refletindo disparidades regionais no acesso aos serviços de vigilância, diagnóstico e tratamento. A qualidade das informações do Sistema de Informação de Agravos de Notificação (SINAN) ainda constitui um desafio, com incompletude superior a 20% nos registros de coinfecção LV/HIV até 2017, o que limita a estimativa real da carga do agravo ^2^. Esse cenário reforça a necessidade de análises atualizadas que integrem dimensões temporais, clínicas e geográficas, de modo a subsidiar ações de vigilância mais sensíveis às desigualdades regionais.

Com base nesse contexto, este estudo analisou a tendência temporal e o perfil clínico-epidemiológico da coinfecção por LV/HIV nos estados brasileiros com maior incidência de LV entre 2009 e 2024, utilizando como indicador o coeficiente anual de coinfecção LV/HIV (por 100 mil habitantes). O objetivo é oferecer evidências concretas que subsidiem ações de vigilância mais sensíveis às desigualdades regionais e sociodemográficas observadas.

## Metodologia

Trata-se de um estudo ecológico de série temporal e transversal descritivo, baseado em dados secundários do SINAN, referentes aos casos de LV e coinfecção LV/HIV notificados entre 2009 e 2024. Foram incluídos os estados com maior número de casos de LV no período: Bahia, Ceará, Maranhão, Minas Gerais, Mato Grosso do Sul, Pará, São Paulo e Tocantins. Os dados foram obtidos no sítio eletrônico do Departamento de Informação e Informática do Sistema Único de Saúde (DATASUS; https://datasus.saude.gov.br/), por meio do programa TabWin (http://www2.datasus.gov.br/DATASUS/index.php?area=060805), valendo-se da base “LEIV - Leishmaniose Visceral”, no período de dezembro de 2024 a fevereiro de 2025. As estimativas populacionais foram obtidas do Instituto Brasileiro de Geografia e Estatística (IBGE; https://www.ibge.gov.br/).

A variável de interesse foi o coeficiente anual de coinfecção LV/HIV, calculado pela razão entre o número de casos notificados de coinfecção LV/HIV e a população residente no respectivo estado no mesmo ano, multiplicada por 100 mil. A tendência temporal foi analisada por meio de regressão segmentada, utilizando o programa Joinpoint (https://surveillance.cancer.gov/joinpoint/). Foram estimadas a variação percentual anual (APC, acrônimo em inglês) e a variação percentual média anual (AAPC, acrônimo em inglês), com intervalo de 95% de confiança (IC95%) e nível de 5% de significância.

As demais análises estatísticas foram realizadas no software IBM SPSS, versão 27 (https://www.ibm.com/). A normalidade das variáveis contínuas foi testada pelos métodos de Shapiro-Wilk e Kolmogorov-Smirnov. A correlação entre o número de casos de LV e de coinfecção foi investigada pelo teste de Spearman, utilizado aqui com o objetivo de verificar a consistência da relação monotônica esperada. A comparação das médias de idade entre os grupos foi realizada pelo teste t de Student para amostras independentes, com verificação da homogeneidade de variâncias pelo teste de Levene. O tamanho do efeito foi estimado pelo d de Cohen. Por se tratar de estudo censitário, esse indicador tem caráter exclusivamente descritivo, servindo para expressar a magnitude da diferença observada, e não para inferência amostral.

As associações entre variáveis categóricas (sexo, raça/cor, escolaridade e conduta terapêutica) foram analisadas pelo teste qui-quadrado de Pearson. Para identificar sintomas clínicos associados à coinfecção (febre, fraqueza, edema, emagrecimento, tosse e/ou diarreia, palidez, esplenomegalia, infecção associada, hepatomegalia e hemorragias), utilizou-se regressão logística binária, com exclusão dos casos com dados faltantes. As associações foram expressas por razão de chances (*odds ratio* - OR), com IC95%.

A qualidade dos dados foi avaliada segundo o critério previamente descrito ^10^, que classifica o grau de completude conforme o percentual de incompletude dos campos: excelente (< 5%), bom (5-10%), regular (11-20%), ruim (21-50%) e muito ruim (> 50%). Foram considerados incompletos os registros em branco ou preenchidos com a opção “ignorado”. Dos 115.731 registros de LV incluídos no banco de dados, 65.757 (56,8%) apresentavam informações completas sobre os sintomas clínicos analisados e foram incluídos na regressão logística. Os 49.974 casos (43,2%) com dados faltantes, em pelo menos uma variável independente, foram excluídos automaticamente pelo método *listwise deletion* do SPSS, assegurando a consistência do modelo.

Por se tratar de dados públicos, de acesso irrestrito e sem identificação individual, o estudo foi dispensado de apreciação pelo Comitê de Ética em Pesquisa, conforme a Resolução nº 510/2016, do Conselho Nacional de Saúde.

## Resultados

A análise de completude indicou qualidade excelente para sexo, idade, diagnóstico parasitológico e imunológico; boa para raça/cor; ruim para coinfecção HIV e tratamento inicial; e muito ruim para escolaridade. Os valores detalhados estão na Tabela 1.

**Tabela 1:** Grau de completude das variáveis utilizadas no estudo de leishmaniose visceral (LV) e coinfecção LV/HIV nos oito estados brasileiros com maior incidência de LV 2009-2024.

Variável	% de incompletude	Classificação *	Observações
Sexo	0,01	Excelente	Campo obrigatório no SINAN
Idade	0,00	Excelente	Completude total
Raça/Cor	7,30	Bom	Boa completude, mas com 7,3% de omissão
Escolaridade	95,60	Muito ruim	Alta incompletude (“ignorado/não se aplica”)
Coinfecção HIV	32,60	Ruim	Um terço dos registros sem informação
Diagnóstico parasitológico	4,60	Excelente	Inclui casos “não realizados”
Diagnóstico imunológico (imunofluorescência indireta)	4,60	Excelente	Inclui casos “não realizados”
Tratamento inicial	36,00	Ruim	Alta ausência de registro terapêutico

SINAN: Sistema de Informação de Agravos de Notificação.

Fonte: SINAN (https://datasus.saude.gov.br/).

* Classificação do grau de completude segundo o critério proposto por Mendes et al. ^10^, que categoriza os percentuais de incompletude em: excelente (< 5%), bom (5-10%), regular (11-20%), ruim (21-50%) e muito ruim (> 50%).

A análise de tendência temporal das taxas de coinfecção LV/HIV revelou comportamentos heterogêneos entre os estados com maior incidência de LV (Tabela 2). Observou-se tendência crescente e sustentada na Bahia e no Pará, com incrementos anuais ao longo de todo o período analisado. No Ceará, identificou-se um comportamento oscilante, com elevação significativa entre 2014 e 2018, precedida e seguida por períodos de estabilidade e declínio, respectivamente. O Maranhão apresentou aumento até 2016, seguido de estabilização nas taxas nos anos subsequentes. Em Mato Grosso do Sul, a série temporal evidenciou flutuações marcadas, com alternância entre períodos de crescimento (2009-2014 e 2018-2024) e redução intermediária (2014-2018). Minas Gerais apresentou aumento inicial até 2014, seguido de queda nos anos seguintes, padrão semelhante ao observado em São Paulo, ainda que com redução de menor magnitude e estacionária no segundo segmento. Por fim, Tocantins exibiu três segmentos distintos, com crescimento acentuado no início da série, estabilidade intermediária e novo incremento a partir de 2016.

**Tabela 2:** Análise temporal da taxa de coinfecção leishmaniose visceral (LV)/HIV nos oito estados brasileiros com maior incidência de LV, 2009 a 2024.

Estados brasileiros	Período	IC95%	APC	Classificação
Bahia	2009-2024	0,6; 9,0	4,2 *	Crescente
Ceará	2009-2014	-37,2; 14,5	-4,7	Estacionária
	2014-2018	16,3; 78,2	40,4 *	Crescente
	2018-2024	-24,9; -5,6	-13,2 *	Decrescente
Maranhão	2009-2016	17,4; 58,2	25,4 *	Crescente
	2016-2024	-4,4; 8,1	3,7	Estacionária
Mato Grosso do Sul	2009-2014	3,6; 53,2	12,4 *	Crescente
	2014-2018	-35,5; -2,9	-16,4 *	Decrescente
	2018-2024	10,2; 53,3	19,7 *	Crescente
Minas Gerais	2009-2014	3,7; 29,4	11,3 *	Crescente
	2014-2024	-11,0; -4,4	-6,9 *	Decrescente
Pará	2009-2024	6,4; 16,5	10,4 *	Crescente
São Paulo	2009-2014	2,2; 39,2	9,4 *	Crescente
	2014-2024	-15,1; 0,7	-1,7	Estacionária
Tocantins	2009-2013	22,9; 132,9	41,9 *	Crescente
	2013-2016	-27,5; 0,3	-17,5	Estacionária
	2016-2024	3,2; 34,6	8,4 *	Crescente

APC: variação percentual anual; IC95%: intervalo de 95% de confiança.

Fonte: Sistema de Informação de Agravos de Notificação (SINAN; https://datasus.saude.gov.br/), 2009-2024.

Nota: as tendências foram classificadas como crescentes (APC positiva e p < 0,05), decrescentes (APC negativa e p < 0,05) ou estacionárias (p ≥ 0,05).

* Tendência estatisticamente significativa (p < 0,05).

De modo geral, os resultados da Tabela 2 indicam padrões temporais dinâmicos, com tendência geral de crescimento nas regiões Norte e Nordeste e comportamentos oscilantes ou decrescentes nos estados do Sudeste e Centro-oeste.

**Figura 1:**
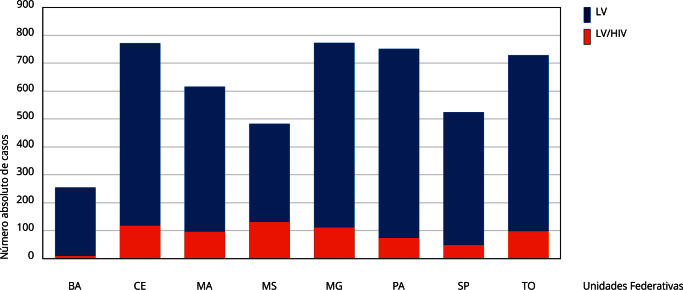
Número absoluto de casos de leishmaniose visceral (LV) e coinfecção LV/HIV segundo Unidades Federativas, Brasil, 2024.

Os coeficientes anuais foram complementados pela análise dos números absolutos de casos de LV e coinfecção LV/HIV por estado, considerando o ano mais recente da série (Figura 1). Essa abordagem combinada permite avaliar simultaneamente a magnitude absoluta das infecções. Em 2024, Mato Grosso do Sul registrou o maior número absoluto de coinfectados (132), seguido por Ceará (119) e Minas Gerais (112). Tocantins (99), Maranhão (97) e Pará (75) vieram na sequência, enquanto São Paulo (49) e Bahia (10) apresentaram os menores valores. Embora os coeficientes revelem padrões distintos entre os estados, a carga absoluta de casos ainda é fortemente influenciada pela incidência geral de LV.

Conforme a Tabela 3, observou-se predominância do sexo masculino em ambos os grupos, mais acentuada entre os coinfectados por LV/HIV (72,4% *vs*. 59,8%; p < 0,001). A maioria foi classificada como parda, mas a proporção de indivíduos pretos foi maior entre os coinfectados (8,8% *vs*. 7%; p < 0,001). Embora a escolaridade tenha apresentado associação com o grupo diagnóstico (p < 0,001), este resultado deve ser interpretado com cautela, uma vez que mais de 95% dos registros apresentaram campos ignorados ou não aplicáveis. Assim, a variável foi mantida apenas por transparência analítica, sem implicações interpretativas.

**Tabela 3:** Características sociodemográficas dos casos de leishmaniose visceral (LV) e coinfecção LV/HIV nos oito estados brasileiros com maior incidência de LV, 2009-2024.

Variável/Categoria	LV	LV/HIV	Teste/Valor
	n (%)	n (%)	
Idade (anos) [Média ± DP]	27,31 ± 24,68	37,98 ± 13,99	t(17,370) = 61,31; p < 0,001; d = 0,45
Sexo			χ^2^ = 517,8 (g.l. = 1) *
Feminino	27.794 (40,2)	2.431 (27,6)	
Masculino	41.415 (59,8)	6.372 (72,4)	
Raça/Cor			χ^2^ = 92,3 (g.l. = 4) *
Branca	12.279 (18,8)	1.383 (17,1)	
Preta	4.594 (7,0)	715 (8,8)	
Amarela	531 (0,8)	40 (0,5)	
Parda	47.508 (72,5)	5.942 (73,4)	
Indígena	576 (0,9)	18 (0,2)	
Escolaridade			χ^2^ = 75,8 (g.l. = 4)*
Analfabeto	123 (0,2)	7 (0,1)	
Até o Ensino Fundamental	1.717 (2,5)	111 (1,3)	
Até o Ensino Médio	987 (1,4)	80 (0,9)	
Até o Ensino Superior	181 (0,3)	14 (0,2)	
Ignorado/Não se aplica	66.206 (95,7)	8.592 (97,6)	

d: tamanho do efeito de Cohen; DP: desvio padrão; g.l.: graus de liberdade; t: teste t de Student; χ^2^: teste qui-quadrado de Pearson.

Fonte: elaboração própria, com base em dados do Sistema de Informação de Agravos de Notificação (SINAN; https://datasus.saude.gov.br/), 2009-2024.

* p < 0,001: diferença estatisticamente significativa.

A média de idade foi significativamente maior entre os coinfectados (37,98 ± 13,99 anos) em comparação aos casos de LV isolada (27,31 ± 24,68 anos; p < 0,001), com diferença média de 10,67 anos e tamanho de efeito moderado (d = 0,45) (Tabela 3). A proporção de homens foi maior entre os coinfectados (72,4%) em comparação ao grupo com LV isolada (59,9%) (χ^2^(1) = 529,81; p < 0,001), resultado esperado diante do predomínio masculino historicamente descrito para a coinfecção LV/HIV.

A regressão logística binária identificou sintomas associados à coinfecção LV/HIV (Tabela 4). Pacientes com febre, icterícia, edema, esplenomegalia e palidez apresentaram maior chance de coinfecção. Em contrapartida, emagrecimento, infecção associada, tosse e/ou diarreia, hemorragias e hepatomegalia estiveram relacionados à menor chance de coinfecção. A variável fraqueza não se mostrou relacionada ao desfecho.

**Tabela 4:** Variáveis clínicas associadas à coinfecção leishmaniose visceral (LV)/HIV em oito estados brasileiros com maior incidência de LV, 2009-2024 (N = 65.757).

Variável clínica	OR (Exp(B))	IC95%	Valor de p
Febre	2,443 *	2,308; 2,587	< 0,001
Fraqueza	1,062	0,998; 1,130	0,056
Edema	1,560 *	1,460; 1,667	< 0,001
Emagrecimento	0,466 *	0,440; 0,494	< 0,001
Tosse e/ou diarreia	0,683 *	0,649; 0,719	< 0,001
Palidez	1,172 *	1,109; 1,239	< 0,001
Esplenomegalia	1,171 *	1,102; 1,245	< 0,001
Infecção associada	0,622 *	0,587; 0,658	< 0,001
Hemorragias	0,810 *	0,745; 0,881	< 0,001
Hepatomegalia	0,824 *	0,774; 0,876	< 0,001
Icterícia	1,686 *	1,568; 1,814	< 0,001

Exp(B): exponenciação do coeficiente logístico (OR); IC95%: intervalo de 95% de confiança; OR: *odds ratio.*

Fonte: elaboração própria, com base em dados do Sistema de Informação de Agravos de Notificação (SINAN; https://datasus.saude.gov.br/).

Nota: modelo ajustado pelo método Enter. Valores de OR > 1 indicam maior chance de coinfecção LV/HIV; OR < 1 indicam menor chance.

* p < 0,05.

Houve associação entre a coinfecção por HIV e a conduta terapêutica inicial registrada para os casos de LV (χ^2^(3) = 3.581,733; p < 0,001). Entre os 57.500 casos com informação válida, 6.837 apresentavam coinfecção por HIV. Nesses casos, a anfotericina B lipossomal foi o tratamento mais utilizado (36,1%), seguido pela ausência de registro de tratamento (43%). O uso de antimonial pentavalente foi menor nesse grupo (8%). Entre os pacientes sem coinfecção, predominou a ausência de especificação do tratamento (50,2%), seguida pelo uso de antimonial pentavalente (29,1%). A anfotericina B lipossomal foi utilizada em 11,9% desses casos.

## Discussão

A análise da tendência temporal e do perfil clínico-epidemiológico da coinfecção por LV/HIV entre 2009 e 2024 evidenciou padrões temporais distintos entre os estados brasileiros com maior incidência de LV. Observou-se tendência de crescimento nas regiões Norte e Nordeste, enquanto os estados do Sudeste e Centro-oeste apresentaram trajetórias predominantemente oscilantes ou decrescentes. A coinfecção ocorreu principalmente em homens adultos e pessoas autodeclaradas pardas, com média de idade maior do que nos casos isolados de LV. Febre, icterícia, esplenomegalia, palidez e edema estiveram associados à coinfecção, ao passo que emagrecimento, hepatomegalia, hemorragias e diarreia se mostraram menos prevalentes entre os coinfectados. Verificou-se ainda maior uso da anfotericina B lipossomal nesse grupo, em consonância parcial com as recomendações terapêuticas nacionais.

Nos estados do Nordeste - Ceará, Bahia e Maranhão - observam-se padrões distintos, porém convergentes quanto à manutenção da coinfecção em patamares elevados. No Ceará, verificou-se declínio das taxas populacionais após 2018, possivelmente relacionado à ampliação da testagem rápida para HIV e à integração das ações de vigilância implementadas nacionalmente a partir de 2017 ^11^.

A Bahia manteve tendência crescente nas taxas de coinfecção ao longo dos anos avaliados. Estudos anteriores indicam que, desde o período de 2001 a 2010, o estado se consolidou como uma das áreas do Nordeste mais afetadas pela coinfecção LV/HIV no Brasil ^12^. Essa situação tem sido atribuída à urbanização progressiva da LV e à influência de fatores socioeconômicos e de mobilidade populacional, que favorecem a sobreposição de contextos de vulnerabilidade e a disseminação simultânea dos dois agravos ^12^ ^,^ ^13^.

No Maranhão, a coinfecção LV/HIV apresentou tendência crescente entre 2009 e 2016, conforme demonstrado em nossos dados. Esse padrão é compatível com o observado por Ribeiro et al. ^5^, que encontraram taxa média de coinfecção de 8,6% no período de 2009 a 2017, associada à letalidade de 8,1% e ao aumento progressivo da mortalidade por LV na região. Apesar de seguimento estacionário entre 2016 e 2024, o Maranhão se mantém entre os cinco estados com maior número absoluto de registros de coinfecção no país (97 casos em 2024). A estabilidade em patamar elevado indica desafios persistentes para o controle integrado da coinfecção, possivelmente relacionados a determinantes sociais, como pobreza e urbanização desordenada ^10^.

A concentração desses índices elevados nas regiões Nordeste e Centro-oeste pode estar associada a fatores socioambientais - como condições socioeconômicas precárias, dificuldades de acesso ao diagnóstico precoce e alta endemicidade de ambas as infecções ^14^ ^,^ ^15^ - bem como à sobreposição geográfica de LV e HIV nesses territórios, que eleva o risco de coinfecção e impõe desafios às estratégias de prevenção e controle ^12^.

No Mato Grosso do Sul, a magnitude da coinfecção LV/HIV tem sido expressiva. Em Campo Grande, entre 2010 e 2022, foram registrados 344 casos de coinfecção, correspondendo a 27,3% dos casos confirmados de LV no período ^13^. Desses, 75,2% ocorreram em indivíduos HIV positivos com recidiva de LV, o que evidencia maior vulnerabilidade clínica e imunológica. O aumento anual das recidivas entre pessoas vivendo com HIV reforça o impacto da coinfecção sobre a gravidade dos casos e a necessidade de monitoramento e seguimento clínico contínuos para essa população.

O aumento da taxa de coinfecção LV/HIV observado no Mato Grosso do Sul a partir de 2018 pode estar associado à melhoria da detecção e da vigilância laboratorial, mais do que a uma elevação real da transmissão. A ampliação da testagem rápida para HIV nas unidades de referência ^11^ e o uso crescente de testes rápidos para LV no país ^2^ contribuíram para a maior identificação de casos coinfectados, refletindo o fortalecimento da vigilância integrada. Parte desse aumento nas notificações pode, portanto, estar relacionada à melhor capacidade diagnóstica e à adoção de protocolos nacionais de testagem, o que se traduz em maior sensibilidade do sistema para a detecção da coinfecção.

No Tocantins, a taxa populacional de coinfecção LV/HIV apresentou tendência crescente entre 2009 e 2024, com aumento médio anual de 8,4% (p < 0,05). Esse resultado é consistente com evidências prévias que apontam elevação progressiva da coinfecção no estado. Entre 2016 e 2018, Pereira et al. ^16^ identificaram coinfecção em 9,8% dos casos de LV, predominantemente em homens adultos jovens e indivíduos com baixa escolaridade. A convergência desses achados reforça o caráter emergente e persistente da coinfecção na Região Norte, sugerindo que a ampliação da testagem sorológica e as desigualdades socioeconômicas locais influenciam o padrão de crescimento observado.

Em Minas Gerais, a taxa populacional de coinfecção LV/HIV apresentou tendência crescente entre 2009 e 2014, seguida de declínio até 2024. O valor decrescente observado em Minas Gerais pode também estar relacionado à capacidade instalada de vigilância, mas não exclui a hipótese de sub-registro persistente, já que é o 3º estado com maior número absoluto de casos de coinfecção no ano de 2024, o que pode configurar falha no controle efetivo do agravo ^17^ ^,^ ^18^. Além disso, o padrão observado nos dados coincide com o período descrito por Cota et al. ^19^, que identificaram elevada frequência de coinfecção e desfechos clínicos desfavoráveis em pacientes atendidos em Belo Horizonte, com 31% de recidivas e 16% de letalidade. Os autores destacaram que mais de 90% dos casos ocorreram em área urbana, evidenciando a urbanização progressiva da LV e sua sobreposição com o HIV em contextos de vulnerabilidade social.

Estudos comparativos corroboram os achados desta pesquisa. Análise realizada no Estado do Piauí, entre 2007 e 2016, identificou padrões clínicos-epidemiológicos da coinfecção LV/HIV, destacando a necessidade de fortalecimento das ações de vigilância epidemiológica ^20^. Outro estudo conduzido na Região Nordeste indicou o Estado do Maranhão com o maior número de coinfectados, 360 casos entre 2018 e 2022 ^21^. Esses resultados reforçam o papel de fatores socioeconômicos e ambientais na distribuição e elevação das taxas de coinfecção nas regiões Nordeste e Centro-oeste do país.

Observa-se que o comportamento das duas infecções está relacionado ao contexto geográfico e aos processos de urbanização e interiorização. Enquanto a LV expande-se para áreas urbanas, a epidemia do HIV tem se disseminado em regiões rurais ou periféricas ^2^ ^,^ ^21^. No Brasil, a LV é endêmica e sua expansão territorial representa um problema de saúde pública ^6^ ^,^ ^15^. Historicamente restrita a áreas rurais semiáridas do Nordeste, acometendo principalmente crianças desnutridas ^4^ ^,^ ^22^. No entanto, a partir do final da década de 1980, surtos urbanos passaram a ser identificados em grandes cidades do Nordeste e, progressivamente, em outras regiões do país. Esse novo padrão está relacionado à migração populacional, à adaptação do vetor *Lutzomyia longipalpis* ao ambiente peridomiciliar após a mudança do paradigma epidemiológico da LV, de uma comorbidade eminentemente rural para urbana. Índices pluviométricos mais elevados correlacionaram-se temporal e geograficamente com a maior incidência de LV humana, especialmente em áreas próximas ao Oceano Atlântico. A associação entre LV e aumento de chuvas foi relatada em outras regiões do Brasil, favorecendo a consequente expansão da doença ^23^.

A urbanização da LV e a expansão do HIV para áreas rurais configuram fatores associados ao aumento da coinfecção LV/HIV no Brasil, processo descrito desde o final da década de 1990 ^24^. Condições socioeconômicas precárias, migração sazonal e limitações de acesso aos serviços de saúde favorecem a sobreposição espacial das duas infecções. A coinfecção apresenta caráter bidirecional: indivíduos com LV podem receber diagnóstico concomitante de HIV, e pessoas vivendo com HIV têm risco até 2.320 vezes maior de desenvolver LV como infecção oportunista ^24^. Essa interação agrava o prognóstico clínico, favorecendo recaídas e maior carga parasitária, associadas à persistente imunossupressão, baixos níveis de CD4+, menor produção de IFN-γ e aumento da expressão de PD1 em linfócitos T - marcadores preditivos de recorrência identificados em estudos recentes ^25^. Apesar da ampla disponibilidade da terapia antirretroviral no Brasil, o diagnóstico tardio e o desconhecimento do *status* sorológico ainda contribuem para a manutenção de desfechos desfavoráveis ^26^.

Em relação ao perfil sociodemográfico, observou-se predomínio do sexo masculino entre os coinfectados (72,4%) e média de idade superior (37,9 anos) em comparação aos casos de LV isolada (27,3 anos). Esses achados sugerem maior vulnerabilidade de homens adultos jovens, relacionada a fatores comportamentais e ocupacionais e à maior exposição ao HIV. Apesar da redução histórica na razão de sexos entre casos de aids no Brasil - 6:1 em 1989 para 1,6:1 em 2009, fenômeno denominado “feminização da aids” - a predominância masculina na coinfecção persiste, indicando influência de contextos específicos de exposição ao vetor e ao HIV ^27^.

Além dos aspectos comportamentais, hipóteses fisiológicas também têm sido propostas. Fatores hormonais, como a imunomodulação promovida pela testosterona, podem reduzir a resposta Th1 e favorecer a produção de IL-10, contribuindo para uma maior susceptibilidade masculina à LV ^28^. A menor procura por serviços de saúde entre homens também pode atrasar o diagnóstico de ambas as infecções, aumentando o risco de desfechos adversos e justificando o número elevado de casos observados no presente estudo. O perfil epidemiológico da LV continua fortemente associado a condições socioeconômicas precárias e atividades rurais, nas quais há maior exposição ao vetor. A predominância de casos entre homens pode, portanto, refletir ocupações em áreas endêmicas, reforçando a intersecção entre determinantes sociais e biológicos.

A coinfecção por LV/HIV está ainda relacionada a alterações na resposta imune, que agravam o quadro clínico. Estudos apontam transição de um perfil Th1 para Th2, com depleção de células T CD4+ e aumento da expressão do HIV-1 latente ^29^. Esses mecanismos contribuem para maior intensidade de manifestações clínicas e risco de recaída, sendo frequente a ausência de anticorpos anti-*Leishmania* coinfectados, devido à baixa resposta humoral ^7^. A resposta viral e a disfunção imune resultante podem, assim, potencializar a gravidade da coinfecção.

A literatura aponta que a *Leishmania* pode favorecer a replicação do HIV ao promover a diferenciação de monócitos em macrófagos e inibir sua apoptose, contribuindo para o agravamento do quadro clínico. Por outro lado, a presença do HIV também compromete a resposta imune contra a *Leishmania*, facilitando a progressão da infecção ^6^. Embora alguns coinfectados evoluam de forma assintomática, estudos indicam maior risco de manifestações graves da LV em pessoas vivendo com HIV/aids ^30^.

Em relação à variável raça/cor, observou-se maior proporção de indivíduos pretos entre os coinfectados, sugerindo desigualdades raciais na exposição ou no acesso ao diagnóstico ^22^. Excetuando-se o Estado de São Paulo - onde a predominância da população branca pode influenciar esse padrão -, as maiores taxas de coinfecção ocorrem em regiões marcadas por desigualdades sociais e barreiras estruturais ^31^. Dados do Ministério da Saúde revelam que, em 2022, 61,7% dos óbitos por aids ocorreram entre pessoas negras ^26^. Essa tendência reforça a hipótese de que os mesmos fatores que agravam os desfechos do HIV também influenciam a distribuição da coinfecção LV/HIV, evidenciando a necessidade de políticas públicas focadas na equidade racial.

No perfil clínico, os pacientes coinfectados apresentaram menor frequência de sinais clássicos como esplenomegalia, e maior prevalência de sintomas atípicos, incluindo diarreia, dispneia e sangramentos. Esses achados são consistentes com a literatura, como demonstrado em estudos no Maranhão, que apontaram frequência reduzida de hepatoesplenomegalia entre coinfectados e maior ocorrência de manifestações gastrointestinais e respiratórias ^15^ ^,^ ^32^.

Também foram relatadas alterações hematológicas nos coinfectados, como pancitopenia e recidiva, associadas à imunossupressão, toxicidade medicamentosa e coinfecções secundárias ^33^. Em nosso estudo, a recidiva foi frequente entre os coinfectados, alinhando-se ao achado de Graepp-Fontoura et al. ^15^, que relataram recidiva em 36,5% desses casos. Além disso, a febre no episódio inicial foi associada ao maior risco de recidiva ^15^. A mortalidade também foi superior nos pacientes coinfectados, em consonância com estudos prévios que indicam letalidade entre 11,1% e 21,6% neste grupo, contrastando com taxas significativamente menores entre os pacientes com LV isolada ^15^ ^,^ ^32^. Fatores como infecção bacteriana secundária, recidiva e presença de edema foram associados à evolução desfavorável. 

Estudo conduzido por Amaral et al. ^(^ ^34^ ^)^ no Maranhão demonstrou a expansão progressiva da LV para áreas urbanas, com mudança no perfil dos acometidos e predomínio de adultos jovens com quadros clínicos mais graves. Esses achados são compatíveis com os resultados desta pesquisa, que indicam alterações no padrão epidemiológico e clínico da LV em indivíduos coinfectados pelo HIV. Apesar dos avanços na sobrevida de pessoas vivendo com HIV após a introdução da terapia antirretroviral (TARV), estudos indicam que a TARV não previne eficazmente as elevadas taxas de recidiva e mortalidade associadas à coinfecção ^15^ - tendência igualmente observada em nossa análise. Em síntese, a coinfecção modifica significativamente o curso clínico da LV, com manifestações atípicas, maior gravidade, recorrência e risco aumentado de óbito. Esses dados reforçam a importância de estratégias diagnósticas e terapêuticas específicas, especialmente em regiões endêmicas e socialmente vulneráveis.

No que se refere ao tratamento inicial, identificou-se heterogeneidade entre os estados analisados. Nos estados do Ceará, Minas Gerais, Pará e Tocantins, a ausência de registro terapêutico foi prevalente, sugerindo falhas no acesso a medicamentos ou na adesão às diretrizes clínicas. A anfotericina B foi mais utilizada no Maranhão, Mato Grosso do Sul e São Paulo, enquanto o antimonial pentavalente predominou na Bahia. No Maranhão, também houve registro do uso de pentamidina em 12 casos, embora este fármaco não seja amplamente adotado na rotina.

A escolha terapêutica parece refletir fatores como disponibilidade de medicamentos, gravidade dos casos e perfil clínico dos pacientes. Embora a anfotericina B lipossomal seja a recomendação preferencial em pacientes coinfectados, devido à sua menor toxicidade renal, seu alto custo e necessidade de infraestrutura hospitalar limitam o uso em áreas de maior vulnerabilidade. Nessas regiões, alternativas como o antimonial pentavalente acabam sendo mantidas, apesar do perfil de toxicidade e eficácia variável. A decisão terapêutica, portanto, deve considerar fatores como acesso, segurança, custo e capacidade instalada dos serviços de saúde ^35^.

Embora este estudo não tenha avaliado diretamente a utilização de medicamentos ao longo do tempo, é importante reconhecer que mudanças nas políticas e rotinas de diagnóstico de ambas as infecções podem ter influenciado as tendências observadas. Em 2023, a *Portaria nº 1.131* do Ministério da Saúde incluiu o teste imunocromatográfico rápido para LV humana na Tabela de Procedimentos do Sistema Único de Saúde, medida que pode ter favorecido a ampliação da capacidade diagnóstica e do registro de casos. Além disso, a realização rotineira de testagem para HIV em pacientes com LV, recomendada em boas práticas assistenciais, pode ter contribuído para aumentar a identificação da coinfecção nas séries mais recentes. Assim, as variações temporais identificadas podem refletir não apenas mudanças epidemiológicas, mas também avanços institucionais no diagnóstico e manejo clínico da LV e da coinfecção LV/HIV.

Os resultados evidenciam a importância de fortalecer a vigilância, o diagnóstico precoce e o tratamento da coinfecção LV/HIV, sobretudo em regiões com elevada carga da doença e vulnerabilidades sociais. A ausência de padronização nos esquemas terapêuticos e a limitada disponibilidade da anfotericina B lipossomal em alguns estados indicam fragilidades na assistência, que podem comprometer o prognóstico dos pacientes. Tais achados reforçam a necessidade de revisão das práticas clínicas e de investimento na qualificação profissional, além da priorização de políticas públicas que promovam o acesso equitativo ao diagnóstico e ao tratamento.

Este estudo apresenta algumas limitações que devem ser consideradas na interpretação dos resultados. A utilização de dados secundários pode estar sujeita a problemas de subnotificação, inconsistências no preenchimento e ausência de informações relevantes, como status imunológico, uso de TARV ou comorbidades. A indisponibilidade de dados desagregados por município limitou análises mais refinadas do contexto local. Cabe destacar, ainda, que mudanças nos protocolos nacionais de diagnóstico e tratamento da LV/HIV ao longo do período analisado - como a ampliação da testagem e a incorporação da anfotericina B lipossomal como primeira escolha terapêutica - podem ter influenciado a comparabilidade temporal dos dados. Apesar dessas limitações, os resultados obtidos fornecem subsídios importantes para o aprimoramento das ações de vigilância e controle da coinfecção LV/HIV no país.

## Data Availability

As fontes de informação utilizadas no estudo estão indicadas no corpo do artigo.
